# Digital health care service reform and health inequity for older people: a quasi-natural experiment in China

**DOI:** 10.3389/fpubh.2023.1217503

**Published:** 2023-11-07

**Authors:** Xinzhu Qi, Tieying Feng, Renyi Deng

**Affiliations:** School of Public Policy and Administration, Xi'an Jiaotong University, Xi'an, China

**Keywords:** digital health care, health reform, health inequity, older people, quasi-natural experiment

## Abstract

**Objective:**

Addressing health inequity (HI) for older people is a pivotal global public health concern, as it impedes the process of healthy ageing. The digital health care service reform (DHSR) emerges as a progressive public health approach to enhance the health and well-being of older adults by providing comprehensive and equitable medical services. This study elucidates the association between DHSR and HI for older individuals to augment comprehension of DHSR implementation.

**Methods:**

The initiation of the action plan for smart health and eldercare (SHE) in 2017 serves as a quasi-natural experiment. Utilizing data from the China Health and Retirement Longitudinal Study (CHARLS) in 2015 and 2018, a propensity score matching (PSM) method was used to select samples, and a difference-in-differences (DID) regression was used to ascertain the net effect of DHSR on HI for older individuals in China. This methodology mitigates selection bias and segregates the DHSR effect from temporal shifts or other occurrences.

**Results:**

The PSM-DID analysis reveals that DHSR reduced the HI index for older individuals by 0.301 (*p* < 0.01). Heterogeneity analyses indicate that the effect of DHSR was more pronounced in older males (−0.333, *p* < 0.01) than females (−0.251, *p* < 0.05). The impact of DHSR was notably higher for older population in the western (−0.557, *p* < 0.01) and central regions (−0.318, *p* < 0.05) compared to the eastern region, where the relationship was statistically non-significant.

**Conclusion:**

The results demonstrate that DHSR plays a vital role in diminishing HI, fostering inclusive growth in public health. The study underscores the imperative of sustained DHSR endeavours and allocating resources to key older demographics to substantially mitigate HI.

## 1. Introduction

The pervasive health inequity (HI) observed among older people undermines the potential for healthy ageing (1), a fundamental aspect of achieving superior quality of life and a significant marker of national progress (2). Consequently, addressing HI has become a paramount objective within global health policies (3). Notwithstanding the global enhancements in medical and health services that have elevated the health conditions of older adults, marked HI persists between rural and urban senior residents across various socioeconomic strata (4), predominantly in low- to middle-income countries (LMICs). In LMICs like China, a pronounced income-driven bias exists favouring the affluent older community in terms of self-rated health, cognitive functions, and activities of daily living (ADL) (5). In response to these escalating health disparities, there has been a sustained international commitment to harness contemporary solutions, such as ‘digital health care services’ to reform health systems and mitigate HI among older individuals.

Digital health care services (DHS) are new public health practices in the digital age that utilize advanced information and communication technologies to enhance the ability of health providers to give remote and timely patient monitoring, interaction, and management (6). DHS has been reported to have both positive and negative impacts on older patients’ HI in existing papers. Online medical services can notably rectify HI for older people through the equitable and prompt distribution of health-promotion resources (7). However, the uneven distribution of eHealth infrastructure between urban and rural areas substantially influences the utilization of health care services among older adults in varied regions (8). Furthermore, given the requisite electronic proficiency to navigate these platforms, a considerable segment of the older population with limited digital familiarity remains susceptible (9), which exacerbates HI. Do DHSs alleviate HI among older adults? What are the heterogeneous characteristics of the impact on different older groups? These issues deserve further exploration.

## 2. Background

### 2.1. Chinese efforts

In China, a consistent pro-rich HI pattern is evident among older people, indicating that those with deteriorating health predominantly belong to lower socioeconomic status. Analysing the China Health and Retirement Longitudinal Study (CHARLS) data, Liu and Li (10) reported that the inequity in activity daily living (ADL) exceeded mental inequity between 2011 and 2015 in the older community. The former was aggravating and the later was alleviating in the five years. Based on a survey conducted in rural areas by the Ministry of Agriculture of China, Tan et al. (11) derived the health concentration index (CI) for older people in eastern, central, and western rural China, with values of 0.0178, 0.0194 and 0.0143, respectively. These findings underscore the urgency to address HI among older people, as it is pivotal to enhance their feelings of accomplishment, contentment, and security.

Recently, digital health intervention is playing an increasingly important role in the practice of reducing HI in China. As the most populous and fastest-growing LMIC, China has attempted to reduce HI for older people by leveraging advancements in information and communication technologies (ICT) and implementing strategies such as “Healthy China” and “Active Ageing” in the digital era (12). Specifically, the State Council developed guidelines to actively promote the “Internet +” initiative in 2015. Also, an action plan for the smart health and eldercare (SHE) industry has been in motion since 2017 and has two planned phases: 2017–2020 and 2021–2025. Issued by the central government, this plan stipulates guidelines for delivering the most effective DHS of older people management to ensure efficiency. The strategic blueprint of this initiative encompasses four primary components: (a) Ensuring universal accessibility to telemedicine and electronic health management services, (b) Fostering research into pivotal digital technologies, including real-time health condition monitoring and big-data-driven health trend analysis, (c) Promoting the development of critical digital products such as micro intelligent sensors, health management wearables, autonomous health detectors, and residential service robots, (d) Instituting financial subsidies to facilitate the adoption of these innovations. Subsequently, the first batch of SHE application pilot demonstrations were launched, engaging 53 emerging enterprises, incorporating 82 designated streets (or townships), and establishing 19 demonstration hubs across 25 provinces.

Notwithstanding these clear goals and recent achievements of specific pilot, the overall success of the various programs is still unclear given the dearth of policy analysis or evaluation of these programs. Consequently, China is a relevant context that warrants further investigation.

### 2.2. Health inequity for older people

HI is the unfair or unethical resource distribution that results in unequal health outcomes between groups (3, 13). HI for older people exhibits a profound correlation with sociodemographic attributes, encompassing gender, educational levels, income, and residential area (4, 14, 15). Behavioral determinants, including tobacco consumption and alcohol intake, also influence HI (16). Additionally, medical factors, such as health expenditure, distance from hospitals and medical reform policies, further increase HI (17).

Attributable to HI, economic deprivation among older people exhibiting unfavourable health outcomes is anticipated to intensify (18). Case and Deaton (19) suggested that older individuals lacking access to medical resources tend to exhibit negative emotions and engage in medically unsound behaviors. Such patterns indirectly perpetuate the cycle between deteriorated health and poverty. A prevailing consensus in both theoretical and practical realms underscores the necessity of sustained societal initiatives to alleviate HI for older people. Tulchinsky et al. (20) emphasized the potential of fortifying community–public health–hospital collaborations to ensure more equitable health care access for all. Joseph et al. (21) argued funding algorithms that incorporate measures of health equity.

Recent studies typically employ Bivariate Rank Dependent Indices, such as the concentration index (CI) and the Erreygers index, to gauge HI (22, 23). Nevertheless, differences within classes are ignored in methods above due to the strong subjectivity of stratified social class groups. Consequently, modern research has shifted towards using the individual as the primary unit of analysis. Pan and Yang (2) adapted the Recentred Influence Function—Index—Ordinary Least Square Regression Model (RIF-I-OLS), originally proposed by Heckley et al. (24), to dissect the origins of socioeconomic inequality in health. Within the framework developed by Pan and Yang (2), the CI of the health score can be converted to the recentered influence function of the concentration index (RIF-CI) of the same score, facilitating variable coefficient estimations using OLS.

### 2.3. Digital health and health inequity for older people

There is a strong movement promoting digital technologies (e.g., computers, phones, wearables) as a means of strengthening health systems and decreasing HI for older people in the digital age (25, 26). Digital health, defined here as the use of technology solutions to deliver health interventions, helps to engender equitable and rapid health promotion resource sharing to reduce HI (7). Health information such as public knowledge of symptoms of common diseases and professional health support from large hospitals are prone to be transmitted to underdeveloped areas through digital health interventions (27). In August 2004, the first telemedicine system based in a county-level hospital was launched in Jingchuan county, Gansu province. Older patients in Jingchuan county who cannot get to the doctor in time will get tele-diagnosis, teleconsultation and telecare services from Lanzhou No.1 hospital, the China –Japan Friendship hospital in Beijing and Osaka hospital in Japan (28).

However, there is persistent inequitable access to DHSs due to the “digital divide” (29). For example, the COVID-19 response illustrates that inequities can deepen when a population with high health literacy can “shelter in place” with telehealth, while other groups have limited access to resources that afford similar choices (30). Similarly, older individuals in remote areas are prone to inequitable access to digital ICTs due to their limited technical skills, low income, insecure housing, and insufficient health literacy (29), which ultimately exacerbates HI (31). Hence, the way to avoid negative effect by the digital divide is to continuously improve ability to use digital devices among older people (32).

### 2.4. Current study

Recent studies have addressed the dual impacts of DHSs on the health and HI among older people; yet, there remains a paucity of empirical research specifically targeting HI among older adults. Notably, a majority of HI investigations emphasize inter-group disparities rather than intra-group variations. Furthermore, extant health analyses often present a narrow purview, neglecting a comprehensive assessment encompassing physical, psychological, and social participation in public health care. The generalizability of these studies is also questionable.

This study integrates digital health care service reform (DHSR) and HI concerning older individuals within a unified framework, utilizing a quasi-natural experimental design and the CHARLS dataset for empirical examination. We hypothesized that DHSR would diminish HI for older individuals, and investigated this assumption in the study population. Primarily, DHSR enhances HI for older people by facilitating equitable and swift dissemination of health promotion resources. Using health knowledge dissemination as an illustrative case, the advent of portable devices (e.g., mobile phones) enables less healthy older individuals to quickly access information related to symptoms and preventive measures for prevalent diseases, potentially reducing both disease occurrence and treatment durations. Furthermore, augmenting the capabilities of subordinate medical establishments can mitigate potential adverse impacts of the digital divide on the HI for older people, to some degree. Through modalities such as teleconsultation, telediagnosis, and telemonitoring, less-established hospitals in underserved regions can leverage resources from larger institutions, promoting their growth. Consequently, older individuals with lower socioeconomic backgrounds, who may be digitally marginalized, are increasingly likely to access specialized medical services.

Adhering to the WHO’s health definition, we utilized the weighted ratio of the Quality of Well Being Scale (QWB) for physical and mental health and employed RIF-I-OLS to evaluate the present state of individual HI. By treating the action plan for the SHE industry as a quasi-natural experiment, a difference-in-differences (DID) regression is conducted to elucidate the relationship between DHSR and HI for older people. As noted, the initial phase of the action plan for the SHE industry, enacted in 2017, was a national policy, having a significant impact on DHS. This policy facilitated comprehensive access to telemedicine, advocated for research into pivotal digital technologies and products tailored for care of older people, and allocated financial subsidies to support their adoption. Consequently, the SHE action plan provides an avenue to ascertain the effects under investigation.

This study offers several significant contributions: (1) It pioneers the evaluation of the execution of China’s SHE industry through the lens of HI. The central aim of this policy initiative is to harness digital technology in enhancing the health and well-being of older population via the provision of comprehensive and balanced medical services. Consequently, an enhancement in HI for older people serves as a critical metric for assessing the efficacy of digital health policies. The empirical findings presented herein not only provide insights for the refinement of DHSR and public health infrastructure in China but also have implications for advancing health equity and social justice in similar nations. (2) The study delves into the diverse characteristics of DHSR’s health outcomes across various dimensions such as gender and regional disparities, adding depth to the existing body of digital health research. (3) A holistic approach is adopted, encompassing physical function (PF), mental health (MH), and social participation (SP) metrics. The RIF-I-OLS method is employed to compute the individual HI index, offering a methodological benchmark for analogous research endeavors.

## 3. Materials and methods

### 3.1. Data source and study population

Data utilized in this study were sourced from the China Health and Retirement Longitudinal Study (CHARLS), an ongoing national social survey project spearheaded by Peking University. CHARLS seeks to furnish comprehensive and high-quality data concerning the aging process, quality of life, and health statuses of individuals aged 45 and above, catering to the research needs concerning older adults. The inaugural survey commenced in 2011, encompassing 17,708 participants. Selection of participants was accomplished through a multistage, stratified sampling approach. Subsequent follow-ups were executed through face-to-face, computer-assisted personal interviews (CAPI), achieving a response rate exceeding 85% (33).

Given the initiation of SHE application pilot demonstrations in 2017, this study utilizes the two latest waves of data from CHARLS, collected in 2015 and 2018. With the age threshold set at 60 years for the older cohort, individuals below this age were omitted. Older individuals residing in cities where DHSR was introduced in 2018 are categorized as the treated group, while those in cities without DHSR implementation serve as the control group. Missing values for essential variables were imputed using the mean method. Following propensity score matching (PSM), a two-stage panel data set was compiled, comprising 3,027 viable participants: 250 in the treated group and 2,777 in the control group.

### 3.2. Variables

#### 3.2.1. Health inequity

The main dependent variable was HI index for older individuals. This HI index is formulated using three variables: PH, MH, and SP scores. The implementation of DHSR influences the outcomes of PH, MH, and SP among older people. Regarding PH, health management applications allow older individuals to access personal health records, receive medication reminders, and obtain preliminary guidelines to enhance mobility (34), facilitating the prevention and early detection of physiological diseases. In terms of MH, online platforms alleviate negative emotions and enhance MH among older people by offering educational games and health-related short videos (35). Robots for companionship, such as the seal-type robot, Paro, induces positive emotional responses in older adults through tactile interactions (27). Concerning SP, the integration of telemedicine into traditional home health services enables older individuals to prolong their community residency and sustain social interactions (36, 37).

The steps of HI calculation are as follows. The study assessed levels of PF, MH, and SP in older people based on WHO-defined health concepts. The assessment of PF entailed a 9-item questionnaire, querying participants about their capability to independently execute physical tasks such as running, rising from a seat, extending arms, and more. Response codes ranged from 1 (indicating no difficulty in tasks) to 4 (indicating difficulty in all tasks). An aggregate of the 9 responses determined each participant’s PF score, with higher scores indicating diminished PF levels. MH assessment utilized 10 questions derived from the Centre for Epidemiological Studies Depression Scale (CES-D), focusing on participants’ emotional state and behavior over the past week. These questions, incorporated by CHARLS, have demonstrated efficacy (38). Summation of the 10 responses yielded the MH score, where a higher score denotes a lower MH level. Regarding SP, participants addressed 11 items detailing their engagement in various activities during the preceding month. Coded activities included 1 (socializing with friends), 2 (playing Mah-jong or other traditional Chinese games, or frequenting community clubs), 3 (participating in sports or other club activities), and so forth. The cumulative score indicates the diversity of activities in which older adults engaged, with higher scores reflecting enhanced SP levels. To speak of, the indicators we selected were based on dimensions from existing literature (2, 38, 39), which means they are robust. Besides, during the data screening process, we aimed to choose questions that were used to inquire about factual phenomena, avoiding direct subjective health assessments from the respondents. These answers were relatively objective, reducing the subjectivity inherent in self-reported health surveys. Additionally, the indicators above were comprehensive and easy to obtain.

Subsequent to the initial assessment, a comprehensive, tri-dimensional health score was derived for older participants by assigning weights to the three factors according to the QWB’s weighting ratio for physical and mental health. Following this, CI was employed to quantify HI for older people based on health and income levels.

The calculation formula is as follows.


$$CI=\left(h\;|y\right)=\frac{1}{n}\overset{n}{\underset{i=1}{\sum }}\frac{h_{i}}{u}\left(2R_{i}-1\right)$$

Here, $R_{i}$ represents the ranking of income levels throughout the sample. Finally, referring to RIF-I-OLS proposed by Heckley et al. (24), we generated *RIF-CI*, depending on each health score corresponding to income rank. The correlation between CI (group level) and the dependent variable (individual level) was established to estimate the marginal effects of various factors on HI (2). RIF-CI represents the HI faced by older individuals, as the mean of the RIF-CI of all individuals in the sample is equal to the CI of the sample.

#### 3.2.2. Digital health service reform

The principal independent variable of interest in this study is DHSR. The initiation of the SHE industry action plan in early 2017 served as the treatment event with discernible policy impact. In light of the inaugural batch of SHE application pilot demonstrations released at the end of 2017, two dummy variables—policy and time—were established following the DID analysis approach. Participants residing in cities with demonstration streets or bases received a code of 1, otherwise, they were coded as 0. Participants interviewed in 2018, the year after the policy took effect, were coded as 1; otherwise, they were coded as 0. The chief independent variable constituted the interaction term of the policy and time dummy variables.

#### 3.2.3. Control variables

Building on prior research regarding factors influencing HI (40), this study incorporated demographic characteristics, lifestyle habits, and medical attributes as control variables. Demographic characteristics included age, educational level, marriage status, marriage satisfaction, and relationship satisfaction with children. Income level was excluded from the control variables, given its incorporation in the CI calculation formula before executing the regression analysis. Lifestyle habits embraced factors such as smoking, drinking, exercise, hours slept and household hygiene level. Medical attributes encompassed medical cost, hospital distance and medical satisfaction.

#### 3.2.4. Other independent variables for heterogeneity tests

We were also interested in the heterogeneous effects of the DHSR on the HI of older persons of different genders and areas. Sex is coded as 0 (females) and 1 (males). Area is coded as 1 (east), 2 (central) and 3 (west).

### 3.3. Analytic approach

PSM was used to reduce selection bias, which allowed us to mimic some of the characteristics of a randomized controlled trial by balancing the distribution of all the covariates in the treated and control groups. First, we estimated the average treatment effect on the treated (ATT) with a series of matching methods when policy was the dependent variable, RIF-CI was the outcome variable and other variables (listed in Table 1) were controlled to verify the robustness of the PSM approach (presented in Supplementary Material S1). Second, older individuals covered in the first batch of SHCOP application pilot demonstrations were matched at a 1:4 ratio to the controls (41).This ratio suggested that this method could usually obtain the least mean square estimation error in a calliper of 0.00878 (one-fourth of the standard deviation (SD) of the propensity score) (42), which is a trade-off between promoting the weight of the smaller group (the treated group) and keeping a good matching quality. Third, SD in covariates between the treated and control groups before and after matching were calculated to assess the improvement of balance in the covariates. If the SD was <10%, it was considered an indicator of balance. Finally, each weight of all matched samples was recorded, and all the unmatched samples were dropped.

**Table 1:** Description of the key independent variables for the study sample.

	Total (*N* = 3,027)		Control (*N* = 2,777)		Treated (*N* = 250)	
Variable	Mean/%	SD	Mean/%	SD	Mean/%	SD
Policy	26.5		19.91		100	
Time	30.7		24.45		100	
Treatment = Policy*Time	8.26		0		100	
Age	69.49	7.441	69.39	7.355	70.62	8.276
Male	47		47.1		45.6	
Educational level	2.995	1.993	2.973	1.998	3.236	1.934
Area						
Dummy east	36.373		36.550		34.4	
Dummy central	35.249		34.930		38.8	
Dummy west	28.378		28.520		26.8	
Marital status						
Dummy married and living with spouse	72.679		73.425		72.4	
Dummy married but not living with spouse temporarily	4.130		3.997		5.6	
Dummy separated, do not live together as a couple anymore	0.198		0.180		0.4	
Dummy divorced	1.024		0.972		1.6	
Dummy widowed	20.548		20.670		19.2	
Dummy never married	0.760		0.756		0.8	
Marriage satisfaction	2.567	0.758	2.562	0.753	2.624	0.803
Parent–child relationship satisfaction	2.314	0.695	2.31	0.696	2.348	0.691
Smoking	89.6		89.1		95.2	
Drinking	35.4		35.6		33.2	
Exercise	92.9		93.4		87.2	
Sleep Hours	6.365	1.978	6.382	1.965	6.175	2.112
Household hygiene level	2.975	0.807	2.977	0.82	2.952	0.651
Medical cost	1,498	1,191	1,503	1,239	1,440	315.7
Medical satisfaction	2.626	1.036	2.623	1.037	2.656	1.034
Hospital distance	29.79	15.38	29.89	15.86	28.63	8.358

Following PSM, a total of 3,027 matched samples with weights were loaded into the dataset of the next several regressions, with 250 samples in the treated group and 2,777 samples in the control group. RIF-I-OLS was adopted to confirm the mitigative effect of DHSR on older HI in China. In this model, we took the mean RIF-CI as the dependent variable to represent the CI of the health score (CI) because the expectation of RIF-CI was equal to CI. Meanwhile, the DID model was used with an aggregation technique to assess the net effect of the policy, which meant that time and policy as well as other variables (listed in Table 1) were all controlled. In addition, gender and area concerned us; thus, we took gender and area as two grouping criteria in the next spatial analysis. All statistical analyses above were performed using Stata 15.1 (Stata Corp., LLC). A *p* value < 0.05 was considered statistically significant.

## 4. Results

### 4.1. Descriptive analysis

The descriptive statistics summarized in Table 1 highlight the diversity of the CHARLS sample after PSM. A total of 47.0% of participants were male. The mean age of the sample was 69.49 years. The average educational level of participants is 2.995, which means that the average participant “did not finish primary school.” The proportions of Eastern and Central participants were similar, 36.373% and 35.249%, respectively. The majority (77.007%) of the sample was married. Of the participants, 89.6% smoked, and 35.4% drank alcohol. The majority (92.9%) of the sample habitually exercised. The mean number of hours slept by the sample was 6.365 h. The average satisfaction with marriage and the parent–child relationship of the participants was 2.567 and 2.314, respectively, which means they were “very satisfied.” The average satisfaction with medical treatment of participants was 2.975, which means they were “somewhat satisfied.”

Table 2 displays the CI values and the mean RIF-CI for participants in both 2015 and 2018. Both CIs (0.0269 and 0.0479) are positive, indicating a pro-affluence HI for older people. This reveals health disparities favoring those with higher incomes, while disadvantaging the less affluent. The 2018 CI (0.0479) exceeded the 2015 value (0.0269), signifying an expanding HI among older individuals between these years.

**Table 2:** Description of health statistics in 2015 and 2018 for the study sample.

	2015 (*N* = 2098)		2018 (*N* = 929)	
Variable	Mean	SD	Mean	SD
Physical function	21.33	2.243	21.56	2.441
Depression	7.763	5.872	7.866	5.477
Activity	0.901	1.141	0.940	1.153
Health score	0.413	0.109	0.416	0.104
Income	18,634	15,460	17,700	21,399
RIF-CI	0.0269	0.598	0.0479	0.615

### 4.2. Baseline results

As we introduced above, PSM-DID was executed, and baseline results were estimated.

**Figure 1:**
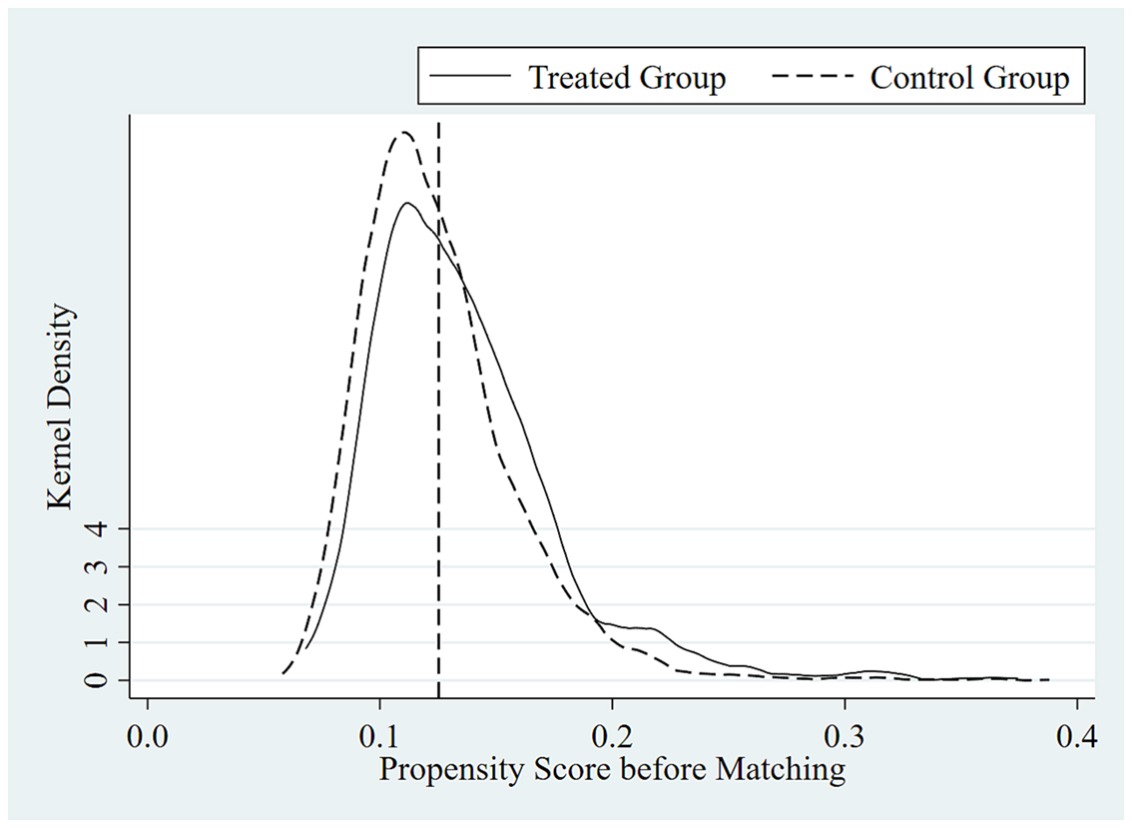
Kernel density function graph of propensity score values before PSM.

**Figure 2:**
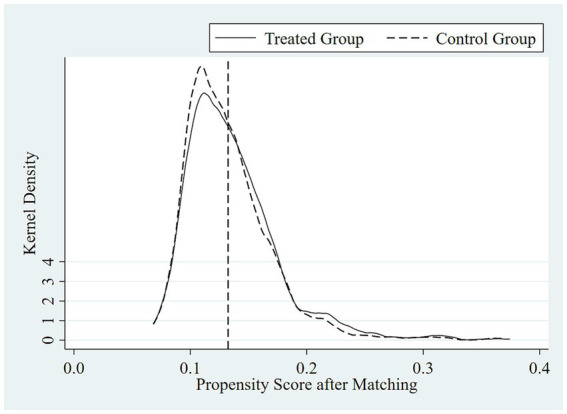
Kernel density function graph of propensity score values after PSM.

**Figure 3:**
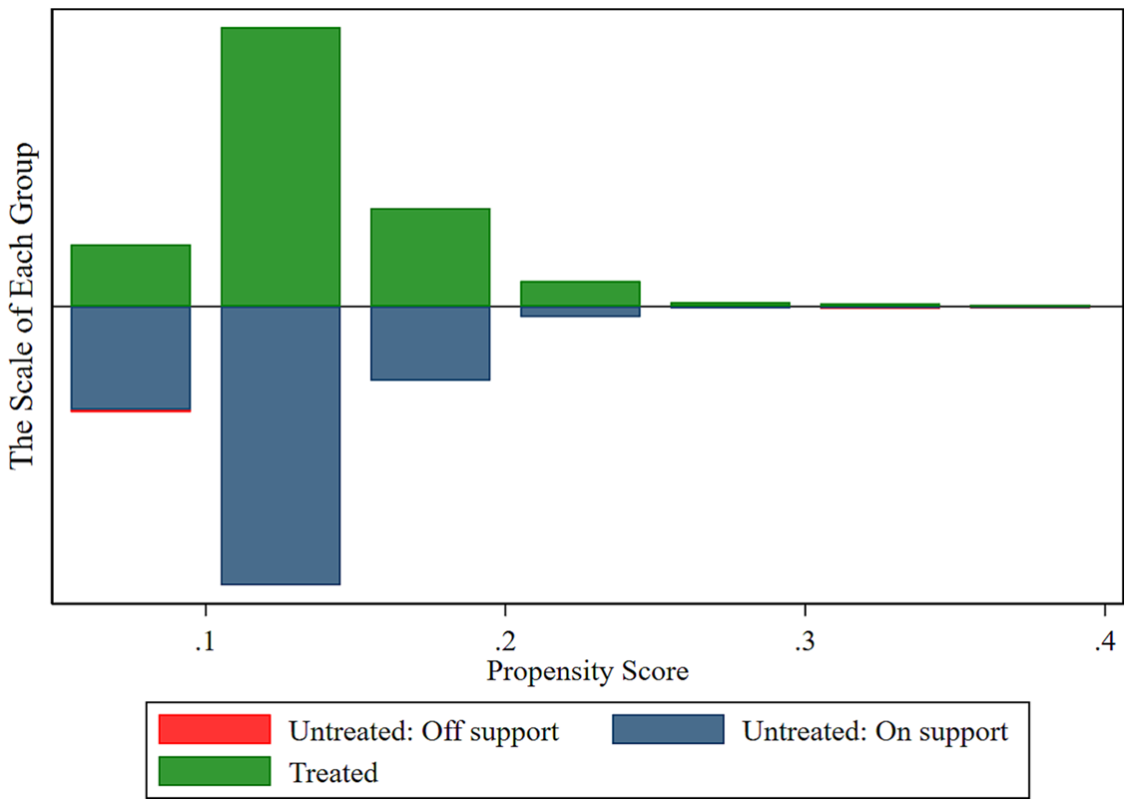
Common support hypothesis test results.

**Figure 4:**
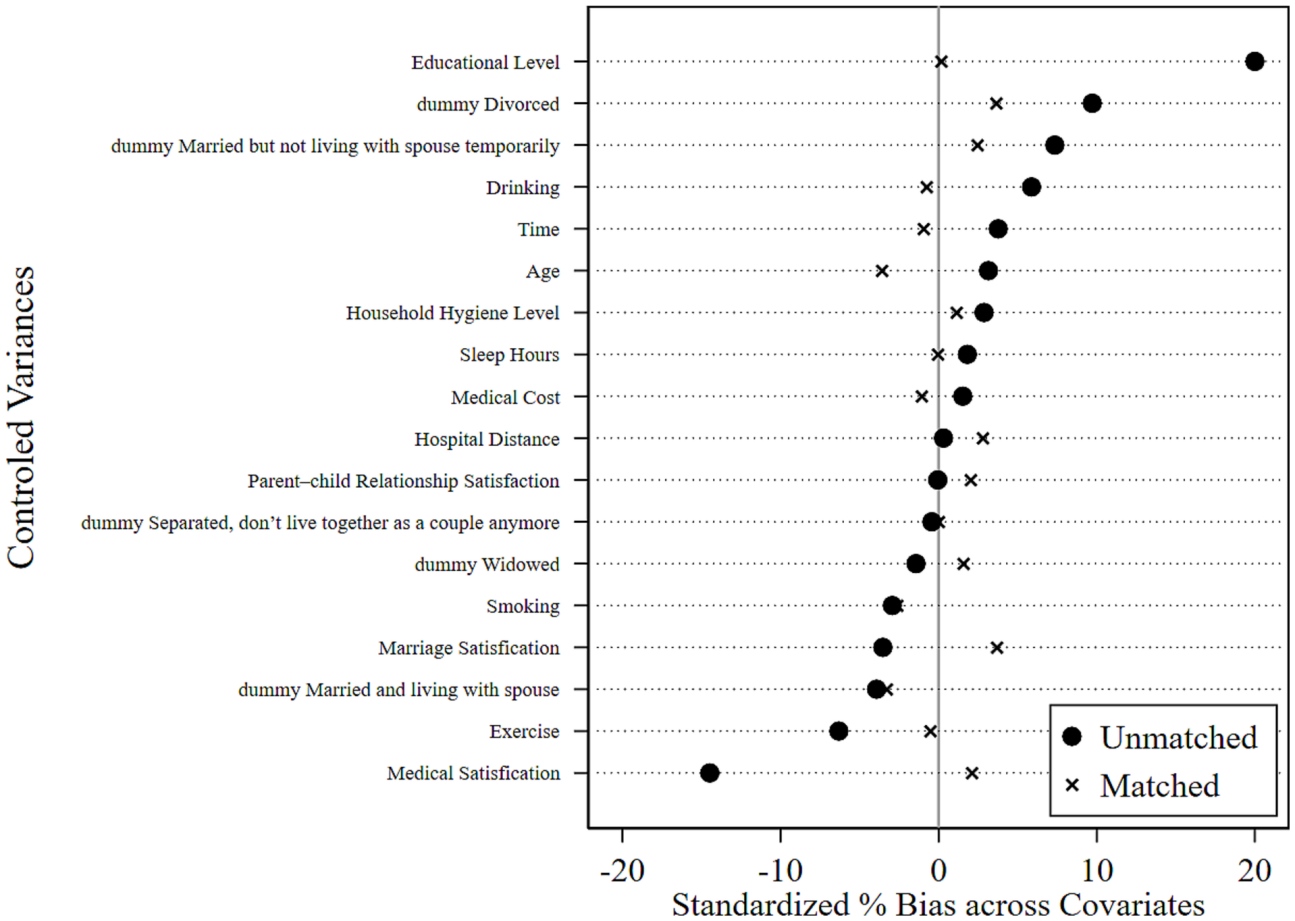
Balance assumption test results.

Figures 1–4 show the optimization effect on the dataset after executing PSM. Figures 1, 2 show the graphs of the kernel density function for propensity score values before and after PSM, respectively. Compared to the gap between the kernel density function curves of the propensity score values between treated and control groups before PSM, the one after PSM was narrower and the two curves were coincident, indicating most of the participants in the treated and control groups would be efficiently matched after applying PSM. Moreover, it was shown while testing the common support hypothesis that a close number of the treated group on support and the control group on support shared each propensity score value along the lateral axis in Figure 3, while in contrast, the scale of shared score values among participants off supports was far less. The data passed the test of the common support hypothesis for PSM. The standard bias of each control variable before and after PSM is visible in Figure 4. After PSM, all the control variables non-significantly differed between the treated and control groups, which meant that the characteristic distributions of the treated group and control group could be efficiently controlled and the balancing assumption test for PSM was passed.

Table 3 reports the estimations related to the DHSR and HI of older individuals.

**Table 3:** PSM – DID results of the DHSR on the HI of older community.

	(1)	(2)	(3)	(4)
	C	C	C	C
Treatment	−0.281^***^	−0.301^***^	−0.299^***^	−0.274^***^
	(−3.40)	(−3.60)	(−3.58)	(−3.25)
CONTROL	N	Y	Y	Y
Male			0.0910^***^	0.0912^***^
			(3.76)	(3.76)
Dummy east				−0.144^***^
				(−5.06)
Dummy central				−0.0247
				(−0.94)
*N*	3,027	3,027	3,027	3,027
*R* ^2^	0.045	0.068	0.070	0.074
adj. *R*^2^	0.044	0.065	0.067	0.071

*t* statistics in parentheses. ^*^p < 0.1, ^**^p < 0.05, ^***^p < 0.01.

In Model 1, while control variables are omitted, both city and year fixed effects are accounted for. Results from Model 1 suggest that the introduction of DHSR effectively mitigates the HI experienced by older populations, with the HI index registering a decline of 0.281 (*p* < 0.01). Model 2, in comparison to Model 1, incorporates additional controls and indicates that DHSR maintains its notable impact on reducing the HI for older individuals, evidenced by a decrement in the HI index by 0.301 (*p* < 0.01). Both Model 1 and Model 2 substantiate the proposed hypothesis. Based on Model 1, the independent variables used to discuss heterogeneity (gender and area) are successively added to Model 3 and Model 4. Notably, the coefficient for DHSR consistently exhibits a negative value in both models (−0.299, *p* < 0.01 and − 0.274, *p* < 0.01). Additionally, based on the findings from Model 3 and Model 4, both gender and area exert significant influence on the HI for older people, establishing them as pivotal parameters for heterogeneity analysis.

### 4.3. Robustness checks

Aiming to verify the conclusion that it was DHSR that accounted for the descent of HI from the control group to the treated group, we designed a placebo test: we drew a random sample for the variable policy and calculated the DID in the new treated (policy) group at different times to observe whether the randomized DID coefficients were concentrated around 0 in the kernel density map and whether their *t* test values significantly deviated from their true values. If the randomized DID coefficients were concentrated around 0 while their *t* test probability values were mostly above 0.01, the robustness test passed. That is, there was no potential confounding effects of other policies or initiatives, which could have influenced the results.

The test procedure encompasses the following steps: Randomize the association between participants and policy. Generate a new treatment group within the RIF-I-OLS regression model. Execute the regression model, capturing the DID coefficients, their associated *t* test values, and respective probability values. Iterate steps 1 through 3 a total of 1,000 times. Construct kernel density plots for both the estimated DID coefficients and their *t* test values, complemented by a scatter plot representing the estimated DID coefficients against their *t* test probability values.

**Figure 5:**
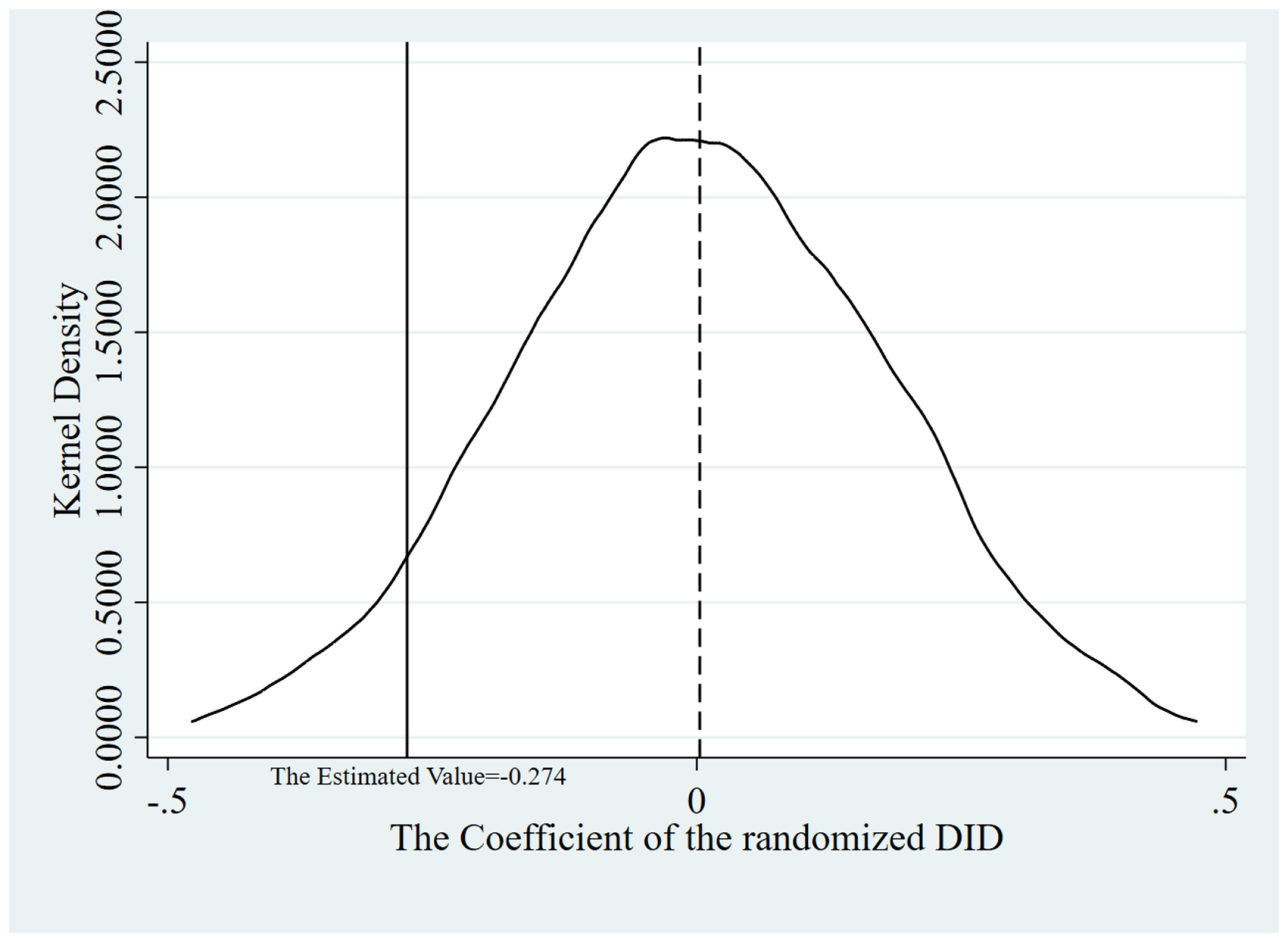
Kernel density diagrams of the estimated DID coefficients.

**Figure 6:**
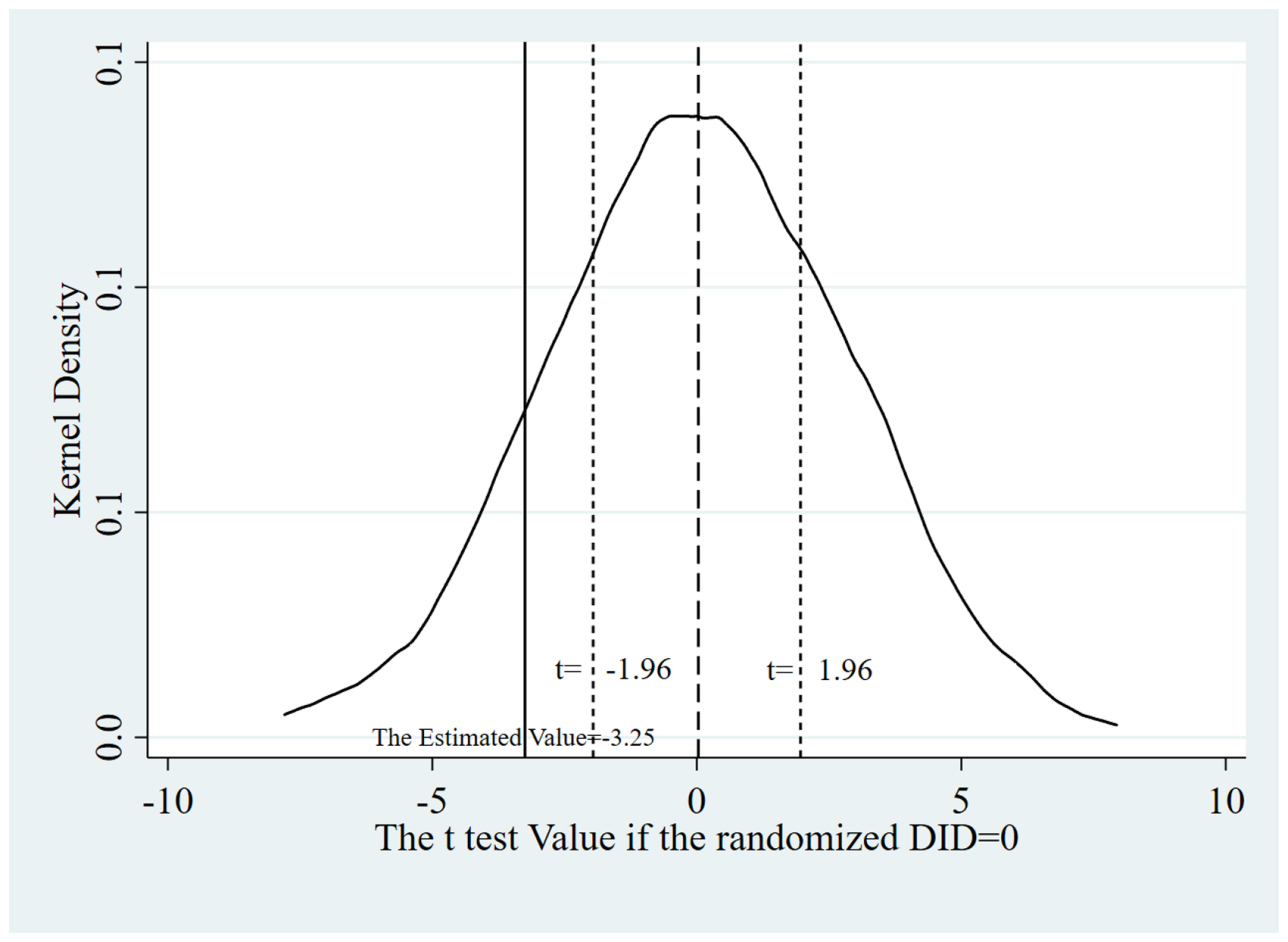
Kernel density diagrams of the estimated DID coefficient and t test values.

**Figure 7:**
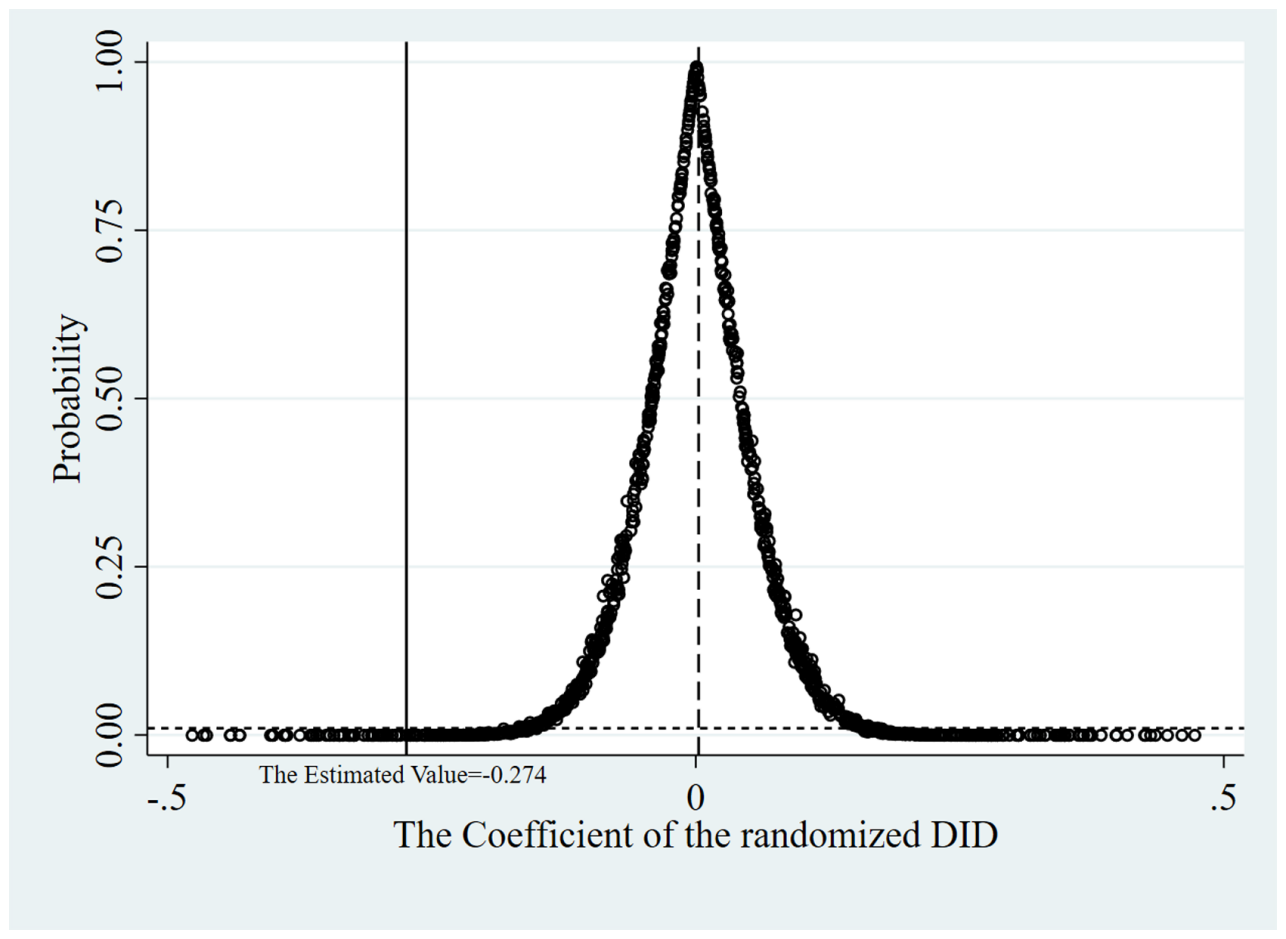
Scatter plot of the estimated DID coefficients and its t test probability values.

Figures 5–7 show that the robustness test passed. When the order of the variable policy, which depended on whether DHSR was executed, the randomized DID coefficients were concentrated around 0 while their *t* test probability values were mostly above 0.01. This indicates that there is a very small probability of misattributing disturbances from unknown variables, including the implementation of other health policies, to the impact of DHSR on HI.

### 4.4. Heterogeneity tests

A detailed examination of the heterogeneity in the impact of DSHR on HI is essential for formulating precise differentiated policies. Table 4 presents the heterogeneity test results, considering gender and regional perspectives. Panel A elucidates the varied effects of the DHSR on male and female populations. Notably, the DHSR substantially alleviates the HI for both older males and females. The influence on older males (−0.333, *p* < 0.01) surpasses that on older females (−0.251, *p* < 0.05). Panel B of Table 4 illustrates the differential impacts of the DHSR on older inhabitants of Eastern, Central, and Western China. The HI index for older people in the western region witnesses a decline by 0.557 (*p* < 0.01), while in the central region, it drops by 0.318 (*p* < 0.05). Contrastingly, the eastern region’s older population exhibits no significant alterations in the HI index. Such findings confirm that the DHSR markedly mitigates the HI for older people in central and western areas, with the impact being more profound in the western than in the central region. Collectively, the DHSR exerts a more significant effect on ameliorating the HI among older demographics facing elevated health risks or residing in less affluent areas, thereby promoting inclusive advancement in social welfare.

**Table 4:** Heterogeneity test results of the DHSR on the HI of older individuals.

(A) Heterogeneity effects by gender			
	Total	Female	Male
Treatment	−0.301^***^	−0.251^**^	−0.333^***^
	(−3.60)	(−2.15)	(−2.76)
CONTROL	Y	Y	Y
*N*	3,027	1,423	1,604
*R* ^2^	0.068	0.055	0.113
adj. *R*^2^	0.065	0.050	0.107

(B) Heterogeneity effects by area				
	Total	East	Central	West
Treatment	−0.301^***^	0.0769	−0.318^**^	−0.557^***^
	(−3.60)	(0.49)	(−2.43)	(−3.95)
CONTROL	Y	Y	Y	Y
*N*	3,027	1,101	1,067	859
*R* ^2^	0.068	0.073	0.110	0.262
adj. *R*^2^	0.065	0.063	0.103	0.254

*t* statistics in parentheses. ^*^p < 0.1, ^**^p < 0.05, ^***^p < 0.01.

## 5. Discussion

Using a large retrospective cohort in China, this study examined the effects of the DHSR on the HI for older individuals, a factor potentially influencing healthy aging. In contrast to many prior studies that solely concentrated on the direct outcomes of PF or MH post enhancements in digital health access, this study embraced the tri-dimensional health definition posited by WHO. The emphasis lay on the nexus between health and digital reform, observed through the lens of social equity. Relying on the CHARLS data, the combined PSM-DID and heterogeneity analysis brought forth several noteworthy insights.

Firstly, the findings highlighted a pronounced negative correlation between the DHSR and HI for older subjects. In the backdrop of rising HI trends among older people, DHSR emerges as a pivotal mechanism to curtail this rise. Contrasting with these findings, certain studies have posited that the proliferation of digital technology does not invariably ensure fair health access, mainly due to the prohibitive cost of high-speed internet for some segments of the population (4, 31). However, the results from other previous studies suggest a conclusion similar to that of this study that digital health tools act as potentially powerful means to alleviate pressure on existing health and social care systems from the point of view of health equity (43–45), especially in the context of COVID-19 (46). Those results are consistent with ours even though much of the evidence is from developed countries and the focus on older populations in developing regions is limited. Our study supplements the literature by examining this association through a quasi-natural experiment on a sample of older people in China.

Digital empowerment may increase the availability of health services and facilitate the flow of health information (44), potentially mitigating disparities in health status. As per Yang et al. (47) suggested that digital health care resources extend beyond any spatial and temporal limitations of hospitals due to their high accessibility, as the Internet of Medical Things (IoMT) increases in popularity. Older individuals, especially those who are relatively poor or in remote areas, are provided lower costs of medical treatment and professional health support through the supply of digital devices and telemedicine (48). The digitization in the public health domain considerably diminishes information transmission costs (49), playing a pivotal role in bridging the chasm in both health information and outcomes (44) essentially amplifying health literacy, thus diminishing the propensity for ailments and mortality. Hence, the momentum towards DHSR remains indispensable.

Furthermore, our study elucidates the diverse impacts of DHSR on HI across various demographic groups. DHSR appears to have a more pronounced influence in mitigating HI among older males and older residing in western and central regions. This observation indicates that the DHSR is more beneficial to older groups with higher health risks or in underdeveloped regions. Digital reforms in public health have led to inclusive growth in social health and well-being. Similar to our conclusions, evidence from developing countries such as Russia and Indonesia showed that the growth generated by digital technology is inclusive, helping to reduce social inequities (50, 51). A study focusing on rural households in China also showed that the digital revolution helped achieve inclusive regional growth (49).

Lin has argued that men suffer higher health risks than women (52). The predisposition of males toward severe chronic conditions and life-threatening diseases stems from their inclination to indulge in health-compromising behaviors such as smoking, alcohol consumption, aggressive confrontations, and substance misuse (53). Dissemination of health-related information, like the hazards associated with smoking, enhances the health literacy among these high-risk groups. This, in turn, motivates them to curtail such perilous habits, thereby narrowing the health disparity gradient through preventive measures. The central and western regions of China, constrained by economic and geographical factors, possess an underdeveloped medical infrastructure, presenting considerable opportunities for enhancement in healthcare accessibility and equity. Here, the DHSR exerts a significant influence in ameliorating health care accessibility and optimizing HI. Conversely, the nonsignificance of impact of DHSR on the HI for older individuals in eastern regions may be attributed to their proximity to superior medical facilities and better medical care.

### 5.1. Policy implications

This study elucidates two pivotal policy implications.

There is a pressing need to expediently advance DHSR initiatives to alleviate HI for older people. Firstly, policy-makers should forge actionable blueprints to incorporate telemedicine into health insurance frameworks, facilitating access for financially constrained older individuals. This could involve initiating pilot programs that center on straightforward, yet mature telemedicine undertakings to catalyze its widespread adoption. Secondly, an imperative exists to supervise the caliber of online health data disseminated. The health care infrastructure should foster the utilization of official online health portals, such as hospital websites, for older individuals seeking credible health information. Concurrently, a personalized educational strategy should be instituted to capacitate older individuals in discerning reliable sources from unreliable ones.

A subsequent recommendation is that policy-makers should identify the core groups whose HI can be substantially mitigated, directing DHSR resources preferentially towards these groups. Such stratification facilitates a distribution pattern aligned with the nationwide Pareto optimality. As a case in point, internet infrastructure development should gain momentum in the western and central regions to ensure seamless remote medical service delivery. The initiation of the “Broadband China” strategy has enhanced broadband accessibility in rural sectors. As of the close of 2018, the internet penetration rate within China’s rural landscapes stood at 38.4%. Concurrently, the broadband transmission speed in these areas witnessed a consistent augmentation, rising from 4 Mbps to 8 Mbps (49). Furthermore, it is essential to emphasize training designed to enhance the digital learning capacity of older individuals.

### 5.2. Limitations

This investigation acknowledges several constraints. Firstly, due to the limitations inherent to the CHARLS dataset, the exclusion of certain potential covariates was necessary, particularly those with extensive missing values, to maintain an expansive sample size. We were unable to derive detailed information about family conditions in childhood (54) and the infrastructural quality of medical establishments—factors potentially influencing health-equity outcomes—remain elusive. Secondly, despite employing PSM analysis to achieve balance among observable confounders, the study could not entirely account for unobserved confounders, thereby allowing the possibility of “hidden bias.” Thirdly, the multi-dimensional intricacies of the DHSR could not be exhaustively explored using the CHARLS data, as the measure was restricted to the action plan for the SHE industry. A more comprehensive exploration of digital reforms might be achieved in subsequent studies by crafting a detailed DHS framework, potentially drawing inspiration from the TIMG digital economy index (55). Additionally, the scope of health metrics related to social participation was limited to 11 activities due to the nature of the CHARLS data. This singular metric could inadvertently narrow the perceived health disparities. Lastly, given the brief interval post-implementation of the SHE industry’s action plan in China, the long-term implications of the DHSR remain undetermined. Future studies should explore these enduring effects and contrast them against the short-term outcomes.

### 5.3. Strengths

Despite inherent limitations, this study boasts four primary advantages. Firstly, it employed PSM analysis to enhance the validity of comparisons—a methodology seldom adopted in prior research. Secondly, to the best of our understanding, this marks the inaugural study probing the health ramifications of the DHSR through the lens of social equity, thereby offering novel insights for policy frameworks fuelling the digital revolution. Thirdly, adhering to the WHO’s tri-dimensional health definition, this research meticulously examined health disparities at an individual level using the RIF-I-OLS technique. Grounded in established literature and theory, this approach addresses an existing void in public health investigations. Lastly, the study relied on comprehensive data that encapsulates China’s entire populace, ensuring the robustness of the findings.

## 6. Conclusion

Utilizing data from CHARLS, this study evaluated the impact of the DHSR on the HI for older individuals in China, offering a potential criterion for digital pilot evaluations. The results revealed that the DHSR played a pivotal role in curbing HI, especially given the rising trend of HI within the older demographic. Notably, the DHSR exhibited pronounced effects on older men and older population in less developed regions, potentially facilitating inclusive advancements in societal health. These findings underscore the imperative of sustaining DHSR efforts and strategically allocating DHSR resources to target older subgroups where significant reductions in HI are attainable.

## Data availability statement

The datasets presented in this study can be found in online repositories. The names of the repository/repositories and accession number(s) can be found below: Publicly available datasets were analysed in this study. This data can be found at: http://charls.pku.edu.cn/.

## Ethics statement

The studies involving humans were approved by All the CHARLS waves was granted from the Institutional Review Board at Peking University. The IRB approval number for the main household survey, including anthropometrics, is IRB00001052-11015; the IRB approval number for biomarker collection, was IRB00001052-11014. The studies were conducted in accordance with the local legislation and institutional requirements. The participants provided their written informed consent to participate in this study.

## Author contributions

XQ and TF researched literature and conceived the study. RD analysed the data. XQ and RD wrote the first draft of the manuscript. TF reviewed and edited the manuscript. All authors contributed to the article and approved the submitted version.

## References

[1] Lee H Kim D Lee S Fawcett J. The concepts of health inequality, disparities and equity in the era of population health. Appl Nurs Res. (2020) 56:151367–5. doi: 10.1016/j.apnr.2020.151367, PMID: 33280788 PMC7521436

[2] Pan C Yang J. The impact of the implementation of hierarchical medical policy on health inequality among th Chinese elderly. Soc Sec Stud. (2022) 80:49–60. (in Chinese)

[3] Asada Y Hurley J OF N Johri M. A three-stage approach to measuring health inequalities and inequities. Int J Equity Health. (2014) 13:98–13. doi: 10.1186/s12939-014-0098-y, PMID: 25366343 PMC4222403

[4] Engelgau MM Zhang P Jan S Mahal A. Economic dimensions of health inequities: the role of implementation research. Ethn Dis. (2019) 29:103–12. doi: 10.18865/ed.29.S1.103, PMID: 30906157 PMC6428178

[5] Gu H Kou Y You H Xu X Yang N Liu J. Measurement and decomposition of income-related inequality in self-rated health among the elderly in China. Int J Equity Health. (2019) 18:4. doi: 10.1186/s12939-019-0909-2, PMID: 30621687 PMC6325844

[6] Ohno T. Capital-labor conflict in the Harrodian model. Evol Inst Econ Rev. (2022) 19:301–17. doi: 10.1007/s40844-021-00199-0

[7] Cresci MK Jarosz PA. Bridging the digital divide for urban seniors: community partnership. Geriatr Nurs. (2010) 31:455–63. doi: 10.1016/j.gerinurse.2010.10.006, PMID: 21188756

[8] Zhang L Han Y. Service model problem and counter measure of smart care for the aged in China. Soc Sec Stud. (2017) 51:30–7. (in Chinese)

[9] Zhan W Xie R. Digital inequalities and social stratification: analysis of the social inequality effect of information communication technologies. Sci Soc. (2020) 10:32–45. doi: 10.19524/j.cnki.10-1009/g3.2020.01.032, (in Chinese)

[10] Liu RP Li JX. Trend and decomposition of health inequality among middle-aged and older adults in China. Popul Dev. (2022) 28:43–55. (in Chinese)

[11] Tan T Zhang Q Liu H. Decomposition of health inequalities among elderly people in rural China:an empirical research based on the survey of eastern， central and Western China. South China Popul. (2015) 30:57–68. (in Chinese)

[12] Lei X Wang J. Intelligent healthy old-age care in the background of “healthy China”: strategic objectives and system construction implementation path. J Northwest Univ. (2020) 50:131–9. doi: 10.16152/j.cnki.xdxbsk.2020-01-013, (in Chinese)

[13] Culyer AJ Wagstaff A. Equity and equality in health and health-care. J Health Econ. (1993) 12:431–57. doi: 10.1016/0167-6296(93)90004-x 10131755

[14] Xue X Ge K. The effect of socioeconomic status on the health of the elderly in China: evidence from the chinese longitudinal healthy longevity survey. Popul Dev. (2017) 23:61–9. (in Chinese)

[15] Sudore RL Mehta KM Simonsick EM Harris TB Newman AB Satterfield S. Limited literacy in older people and disparities in health and healthcare access. J Am Geriatr Soc. (2006) 54:770–6. doi: 10.1111/j.1532-5415.2006.00691.x, PMID: 16696742

[16] Cockerham WC. Health lifestyle theory and the convergence of agency and structure. J Health Soc Behav. (2005) 46:51–67. doi: 10.1177/002214650504600105, PMID: 15869120

[17] Zhao W. Does health insurance promote people's consumption? New evidence from China. China Econ Rev. (2019) 53:65–86. doi: 10.1016/j.chieco.2018.08.007

[18] Jia H. Self-enforcement effect of the “health-related poverty” trap and the endogenous motive force for poverty alleviation: an empirical analysis based on the China family panel survey. Comp Econ Soc Syst. (2020) 4:52–61+146. (in Chinese)

[19] Case A Deaton A. Health and wellbeing in Udaipur and South Africa. In: Wise, DA editor. Developments in economics of aging. Chicago: University of Chicago Press (2009).

[20] Tulchinsky TH Varavikova EA Cohen MJ. Chapter 7 - special community health needs In: Tulchinsky TH Varavikova EA Cohen MJ, editors. The new public health. 4th ed. San Diego: Academic Press (2023). 551–602.

[21] Joseph KT Rice K Li CY. Integrating equity in a public health funding strategy. J Public Health Manag Pract. (2016) 22:S68–76. doi: 10.1097/phh.0000000000000346, PMID: 26599032 PMC5737674

[22] Wagstaff A Paci P van Doorslaer E. On the measurement of inequalities in health. Soc Sci Med. (1991) 33:545–57. doi: 10.1016/0277-9536(91)90212-U 1962226

[23] Erreygers G. Correcting the concentration index. J Health Econ. (2009) 28:504–15. doi: 10.1016/j.jhealeco.2008.02.003 18367273

[24] Heckley G Gerdtham UG Kjellsson G. A general method for decomposing the causes of socioeconomic inequality in health. J Health Econ. (2018) 61:274–4. doi: 10.1016/j.jhealeco.2018.05.005 30049421

[25] Meessen B. The role of digital strategies in financing health care for universal health coverage in low- and middle-income countries. Glob Health Sci Pract. (2018) 6:S29–40. doi: 10.9745/ghsp-d-18-00271, PMID: 30305337 PMC6203415

[26] Confortin SC Corseuil Giehl MW Antes DL Ceola Schneider IJ d'Orsi E. Positive self-rated health in the elderly: a population-based study in the south of Brazil. Cad Saude Publica. (2015) 31:1049–60. doi: 10.1590/0102-311x00132014 26083179

[27] Collins E. Towards robot-assisted therapy: identifying mechanisms of effect in human-biomimetic robot interaction. [Doctoral Thesis, The University of Sheffield]. (2016).

[28] Wang ZL Gu H. A review of telemedicine in China. J Telemed Telecare. (2009) 15:23–7. doi: 10.1258/jtt.2008.080508, PMID: 19139216

[29] Ewing S Rennie E Thomas J. Broadband policy and rural and cultural divides in Australia. In: Digital divides: The new challenges and opportunities of e-inclusion. edited by Andreasson K. Boca Raton: CRC Press. (2015) 107–24.

[30] Jackson DN Trivedi N Baur C. Re-prioritizing digital health and health literacy in healthy people 2030 to affect health equity. Health Commun. (2021) 36:1155–62. doi: 10.1080/10410236.2020.1748828 32354233

[31] Freeman T Fisher M Baum F Friel S. Healthy infrastructure: Australian national broadband network policy implementation and its importance to health equity. Inf Commun Soc. (2019) 22:1414–31. doi: 10.1080/1369118x.2018.1434555

[32] Chen Q Li Y. Bridging digital divide: study on digital skills education for indigenous Australians from the perspective of digital inclusion. In: Studies in Foreign Education. (2023) 50:66–79. (in Chinese)

[33] Chen X Wang Y Strauss J Zhao Y. China Health and Retirement Longitudinal Study (CHARLS). In: Gu D, Dupre M editors. Encyclopedia of Gerontology and Population Aging. Springer, Cham. (2019). doi: 10.1007/978-3-319-69892-2_333-1

[34] Xiang Y Wang X. On the aggregation and reshaping of health management for the elderly in the age of artificial intelligence. Wuhan Univ J. (2020) 73:101–12. doi: 10.14086/j.cnki.wujss.2020.02.010, (in Chinese)

[35] Geng X Zhou Z Wei H Niu G. The impacts of video games on successful aging. Adv Psychol Sci. (2014) 22:295–303. (in Chinese). doi: 10.3724/SP.J.1042.2014.00295

[36] Shi J Wu X Su X. Development of Japan’ s ICT assisted health and ole-age care industry. Res Financial Econ Issues. (2020) 40–8. doi: 10.19654/j.cnki.cjwtyj.2020.06.005.06, (in Chinese)

[37] Chae YM Lee JH Ho SH Kim HJ Jun KH Won JU. Patient satisfaction with telemedicine in home health services for the elderly. Int J Med Inform. (2001) 61:167–73. doi: 10.1016/s1386-5056(01)00139-3 11311671

[38] Zheng C Wang X. The impact of retirement on residents’health: based on regression discontinuity design. Res Econ Manag. (2020) 41:112–28. doi: 10.13502/j.cnki.issn1000-7636.2020.09.008, (in Chinese)

[39] Lu W Pikhart H Sacker A. Domains and measurements of healthy aging in epidemiological studies: a review. The Gerontologist. (2019) 59:294310. doi: 10.1093/geront/gny029 PMC6630160 29897451

[40] Arcaya MC Arcaya AL Subramanian SV. Inequalities in health: definitions, concepts, and theories. Glob Health Action. (2015) 8:27106–6. doi: 10.3402/gha.v8.27106, PMID: 26112142 PMC4481045

[41] Abadie A Drukker D Herr JL Imbens GW. Implementing matching estimators for average treatment effects in Stata. Stata J. (2004) 4:290–311. doi: 10.1177/1536867x0400400307

[42] Rosenbaum PR Rubin DB. The bias due to incomplete matching. Biometrics. (1985) 41:103–16. doi: 10.2307/2530647 4005368

[43] Lyles CR Wachter RM Sarkar U. Focusing on digital health equity. J Am Med Assoc. (2021) 326:1795–6. doi: 10.1001/jama.2021.18459 34677577

[44] Lu J Wang B. Study on the influence mechanism of residents' internet use on their self-rated health: based on China family panel studies in 2016. J Sun Yat-Sen Univ. (2020) 60:117–27. doi: 10.13471/j.cnki.jsysusse.2020.03.013, (in Chinese)

[45] Brodie M Flournoy RE Altman DE Blendon RJ Benson JM Rosenbaum MD. Health information, the internet, and the digital divide. Health Aff. (2000) 19:255–65. doi: 10.1377/hlthaff.19.6.255 11192412

[46] Gunasekeran DV Tseng RMWW Tham Y-C Wong TY. Applications of digital health for public health responses to COVID-19: a systematic scoping review of artificial intelligence, telehealth and related technologies. NPJ Digit Med. (2021) 4:40–6. doi: 10.1038/s41746-021-00412-9, PMID: 33637833 PMC7910557

[47] Yang S Ding S Gu D Li X Ouyang B Qi J. Internet of health care systems (IHS): revolution and innovations of health care management in the new era. J Manag Sci China. (2021) 24:1–11. doi: 10.19920/j.cnki.jmsc.2021.10.001. (in Chinese)

[48] Kong L Hou Y Zheng D. A study of telemedicine policy and patients’ social welfare based on dynamic game. Chin J Manag Sci. (2023) 31:176–86. doi: 10.16381/j.cnki.issn1003-207x.2020.0761. (in Chinese)

[49] Leng X. Digital revolution and rural family income: evidence from China. J Rural Stud. (2022) 94:336–43. doi: 10.1016/j.jrurstud.2022.07.004

[50] Faizah C Yamada K Pratomo DS. Information and communication technology, inequality change and regional development in Indonesia. J Socioecon Dev. (2021) 4:224–35. doi: 10.31328/JSED.V4I2.2669

[51] Skiter NN Ketko NV Rogachev AF Gushchina EG Vitalyeva EM. Institutional poverty as one of the main threats to the digital economy. Int J Sociol Soc Policy. (2021) 41:15–23. doi: 10.1108/ijssp-03-2020-0078

[52] Lin X. Gender trait, body practice and health risk behavior. J Chin Women's Stud. (2011) 20:5–11. (in Chinese)

[53] Courtenay WH. Constructions of masculinity and their influence on men's well-being: a theory of gender and health. Soc Sci Med. (2000) 50:1385–401. doi: 10.1016/s0277-9536(99)00390-1, PMID: 10741575

[54] Bai C Chen D. The origins of health inequality of middle-aged and elderly people in China: measurement and decomposition based on inequality of opportunity. Popul Econ. (2022) 43:104–23. (in Chinese)

[55] Wang Z Chen Y Zhang M. Measuring the development of the global digital economy: stylized facts based on time index. Chin Rev Finan Stud. (2021) 13:40–56. (in Chinese)

