# Supplementary material for: Digital health care service reform and health inequity for older people: a quasi-natural experiment in China
Source: Front Public Health. 2023 Nov 7;11:1217503. doi: 10.3389/fpubh.2023.1217503 (PMC10662057; doi:10.3389/fpubh.2023.1217503)
Supplement: Supplementary file 1 [file Table_1.DOCX]

Supplementary Material

Digital healthcare service reform and health inequity for older people? A quasi-natural experiment in China

Xinzhu Qi, Tieying Feng*, Renyi Deng

*** Correspondence:** Tieying Feng: tyfeng@mail.xjtu.edu.cn

# Supplementary Table

**Supplemental Material Table 1.** Estimated t test Value if ATT=0 in Five Matching Algorithms of PSM

| Matching Algorithm | | ATT=0 T test Value |
| --- | --- | --- |
| K-Nearest Neighbours Matching | K=1 | 5.54 |
|  | K=2 | 5.85 |
|  | K=3 | 5.81 |
|  | K=4 | 6.06 |
|  | K=5 | 6.38 |
|  | K=6 | 6.39 |
|  | K=7 | 6.32 |
|  | K=8 | 6.58 |
|  | K=9 | 6.54 |
|  | K=10 | 6.48 |
|  | K=11 | 6.54 |
|  | K=12 | 6.58 |
|  | K=13 | 6.54 |
| Calliper Matching at 1:4 Radio | Cal=0.0088 | 6.04 |
| Calliper/Radius Matching | Cal=0.0088 | 6.76 |
| Kernel Matching | / | 6.68 |
| Local Linear Regression | / | 4.89 |
